# Supplementary material for: From Gut to Blood: Spatial and Temporal Pathobiome Dynamics during Acute Abdominal Murine Sepsis
Source: Microorganisms. 2023 Feb 28;11(3):627. doi: 10.3390/microorganisms11030627 (PMC10054525; doi:10.3390/microorganisms11030627)
Supplement: Supplementary file 1 [file microorganisms-11-00627-s001.zip › Supplementary-Figure-S3.pdf]

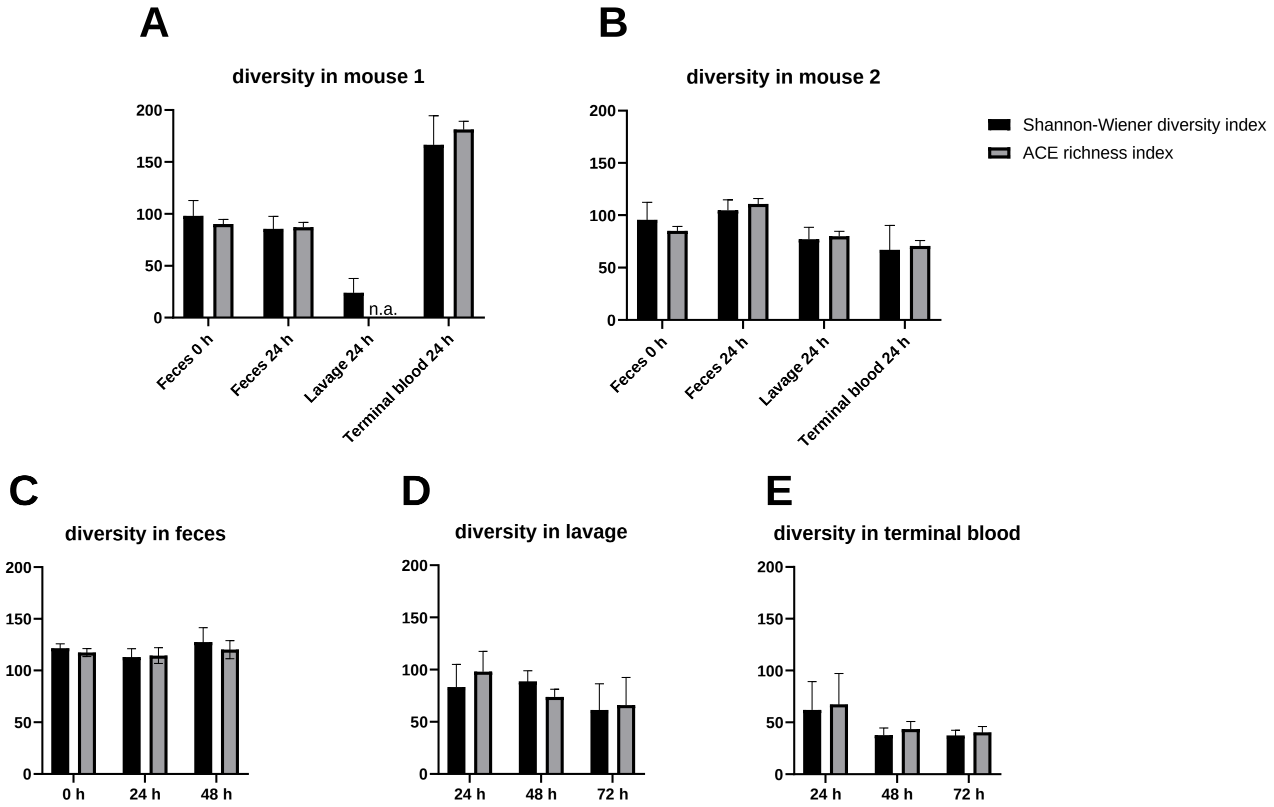
A
diversity in mouse 1
200
150
100
50
0
n.a.
Feces 0 h
Feces 24 h
Lavage 24 h
Terminal blood 24 h
B
diversity in mouse 2
200
150
100
50
0
Feces 0 h
Feces 24 h
Lavage 24 h
Terminal blood 24 h
Shannon-Wiener diversity index
ACE richness index
C
diversity in feces
200
150
100
50
0
0 h
24 h
48 h
D
diversity in lavage
200
150
100
50
0
24 h
48 h
72 h
E
diversity in terminal blood
200
150
100
50
0
24 h
48 h
72 h
