# Supplementary material for: From Gut to Blood: Spatial and Temporal Pathobiome Dynamics during Acute Abdominal Murine Sepsis
Source: Microorganisms. 2023 Feb 28;11(3):627. doi: 10.3390/microorganisms11030627 (PMC10054525; doi:10.3390/microorganisms11030627)
Supplement: Supplementary file 1 [file microorganisms-11-00627-s001.zip › Supplementary-Table-S1.pdf]

Figure	Mouse # in Figure	Unique Mouse ID
2A	1	3805
	2	3806
	3	3807
	4	3809
	5	3810
	6	3811
	7	3812
	8	3813
	9	3814
	10	3815
	11	3816
	12	3818
	13	3819
	14	3820
	15	3822
	16	3823
	17	3824
	18	3826
	19	3827
	20	3828
	21	3829
	22	3830
	23	3831
	24	3832
	25	3833
	26	3834

2B	1	3811
	2	3832
	1	3832
	2	3814
	1	3811
	2	3812
3 and 4	1	3601
	2	3603
	3	3606
	4	3607
	5	3619
	6	3623
	7	3858
	8	3866
	9	3862
	10	3867
	11	3871
	12	3627
	13	3609
	14	3875
	15	3881
5	1	3810
	2	3814
	3	3832
7	1	3876
	2	3880
