# Supplementary figures and images for: From Gut to Blood: Spatial and Temporal Pathobiome Dynamics during Acute Abdominal Murine Sepsis
Source: Microorganisms. 2023 Feb 28;11(3):627. doi: 10.3390/microorganisms11030627 (PMC10054525; doi:10.3390/microorganisms11030627)

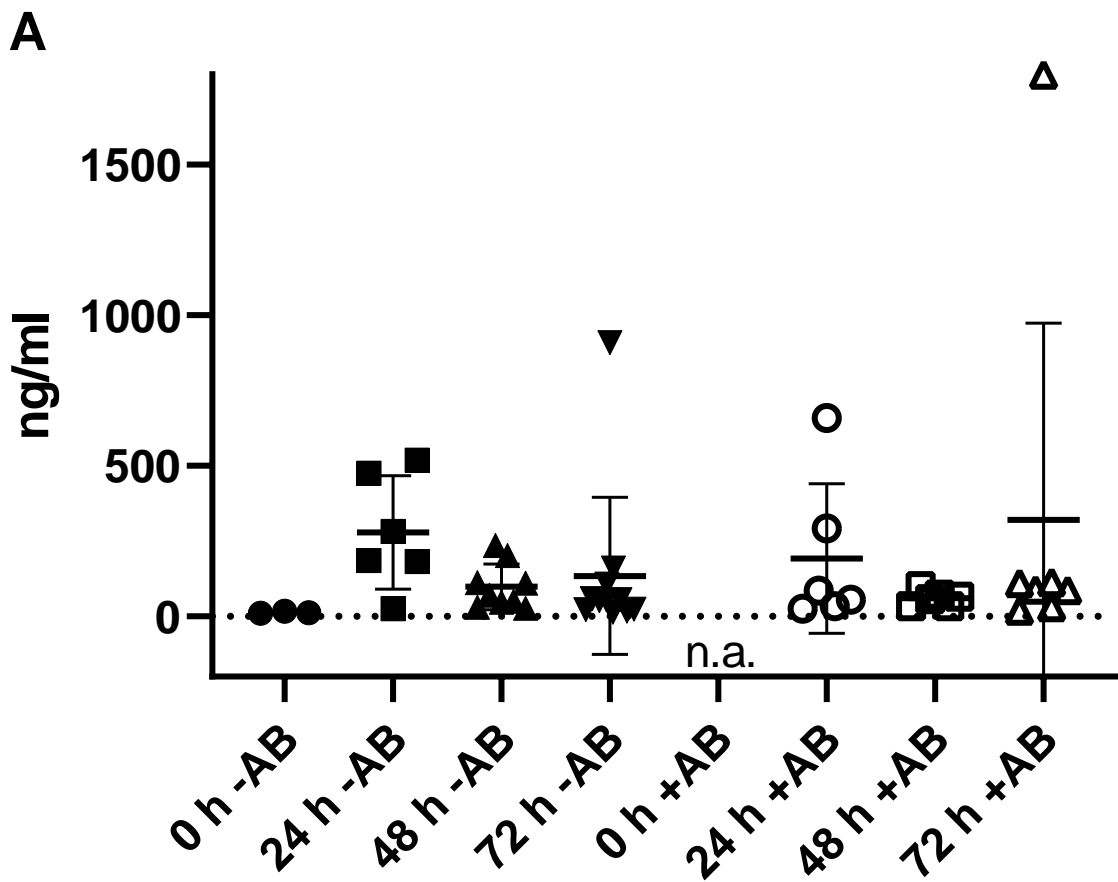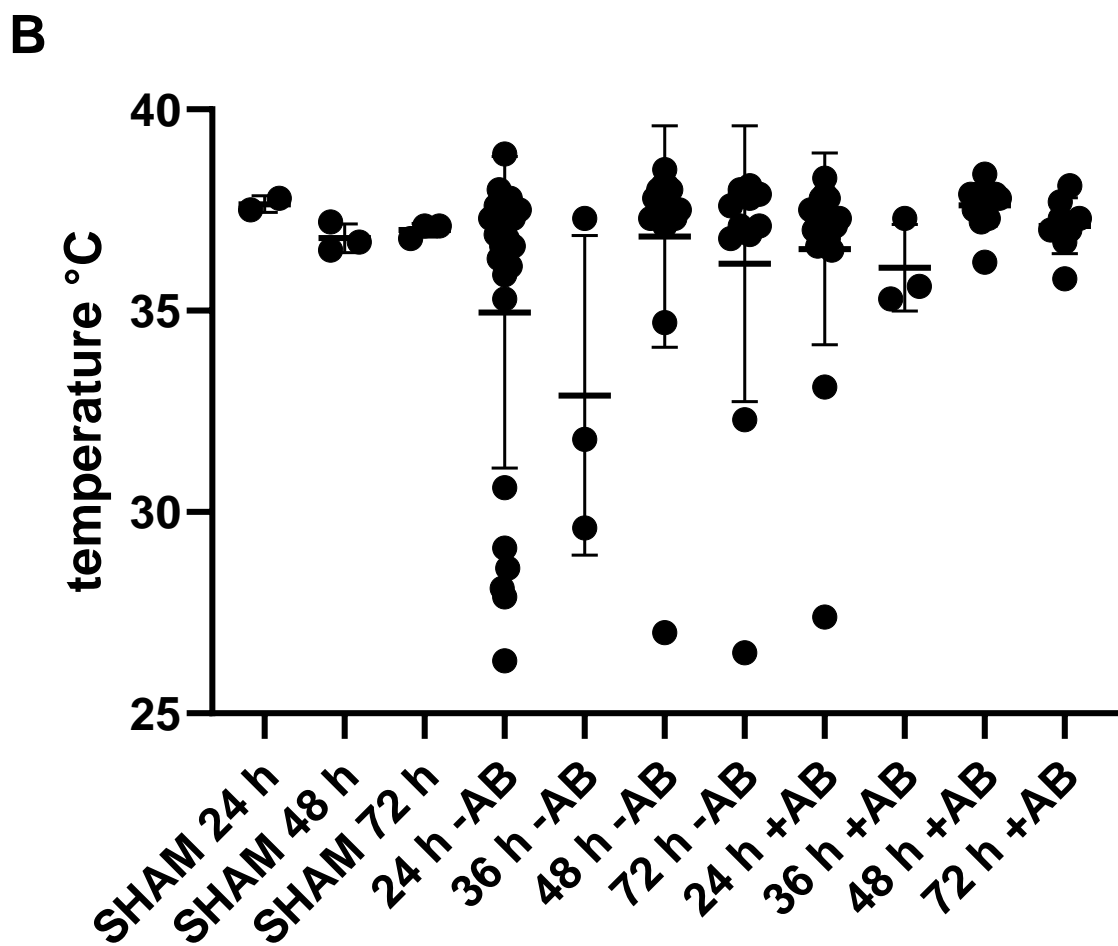

Supplement: Supplementary file 1 [file microorganisms-11-00627-s001.zip › Supplementary-Figure-S1.pdf]

## Slide 1
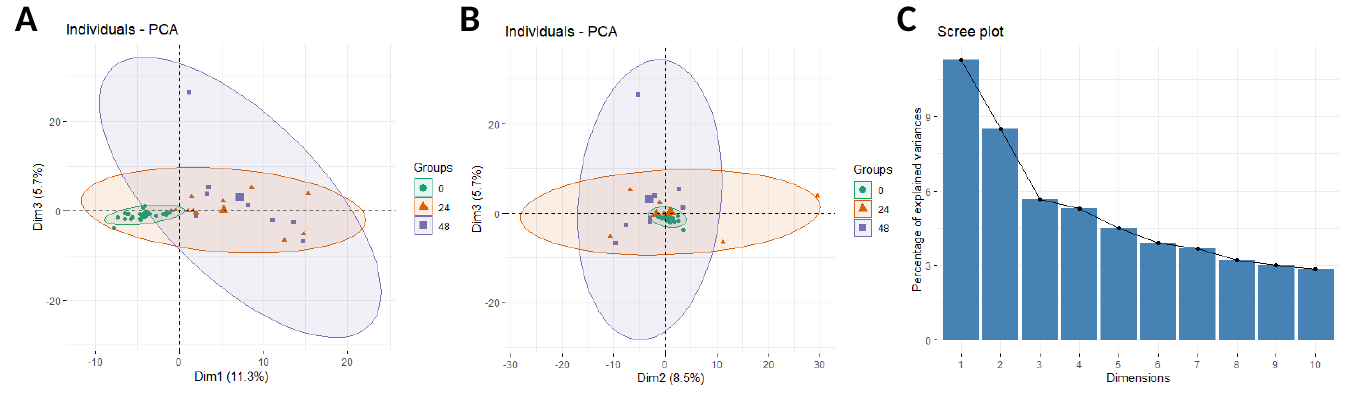

A
B
C

Supplement: Supplementary file 1 [file microorganisms-11-00627-s001.zip › Supplementary-Figure-S2.pptx]

A

## Human

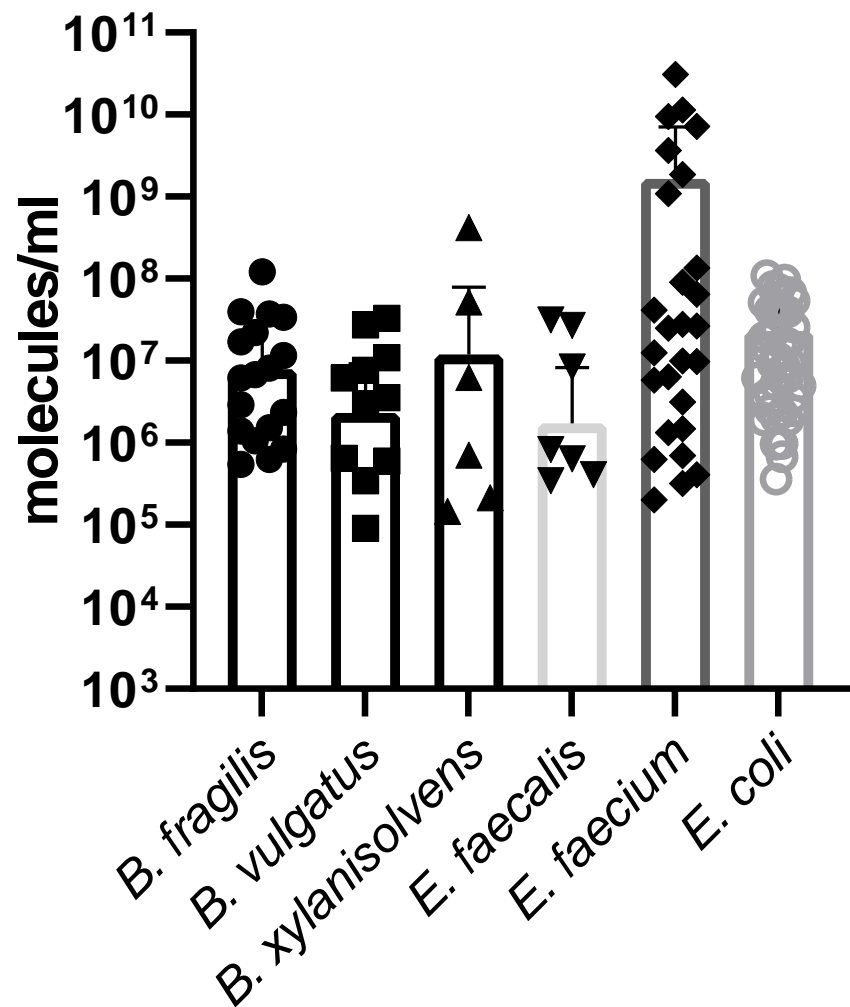

B

## Murine

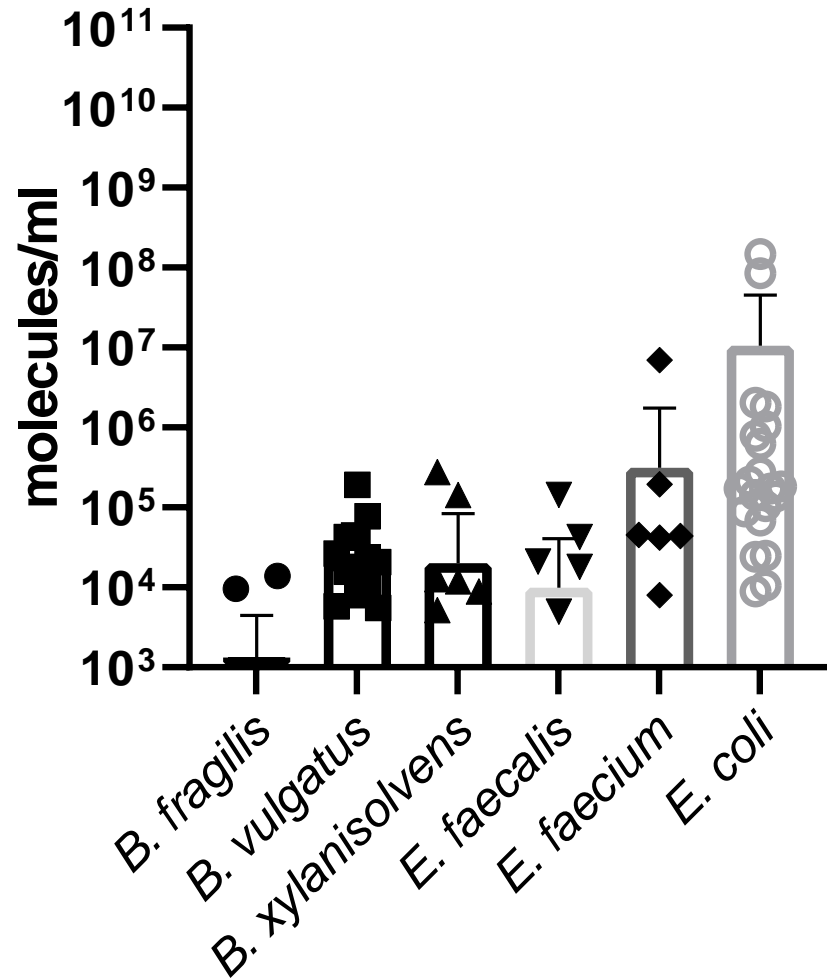

Supplement: Supplementary file 1 [file microorganisms-11-00627-s001.zip › Supplementary-Figure-S4.pdf]

A

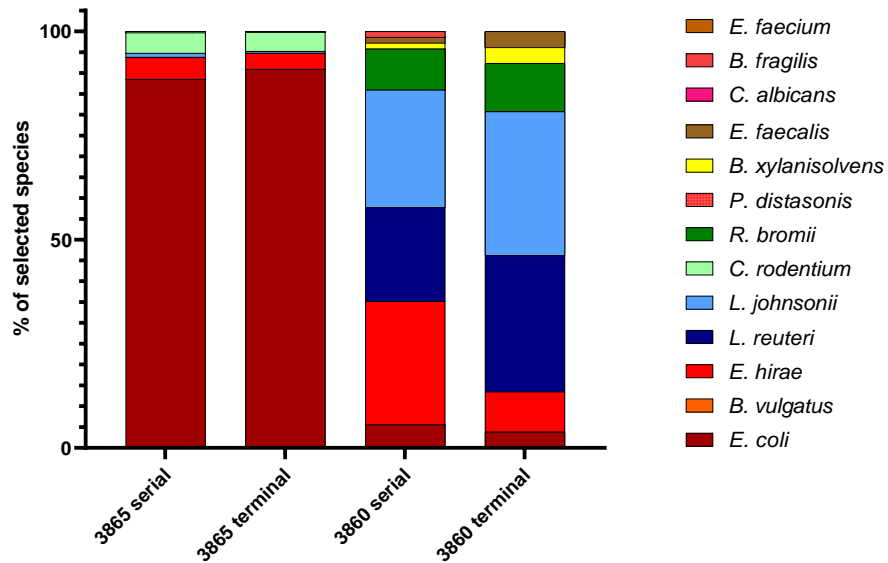

B

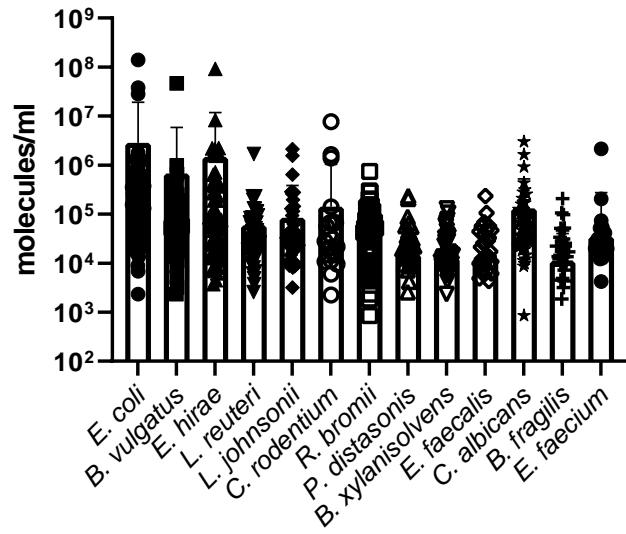

Supplement: Supplementary file 1 [file microorganisms-11-00627-s001.zip › Supplementary-Figure-S5.pdf]

IL-1 $\beta$ 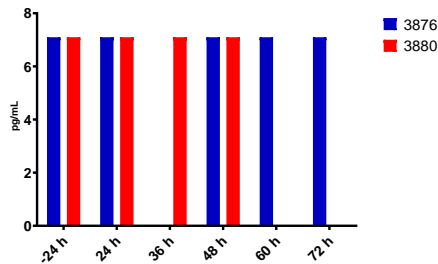

IL-2

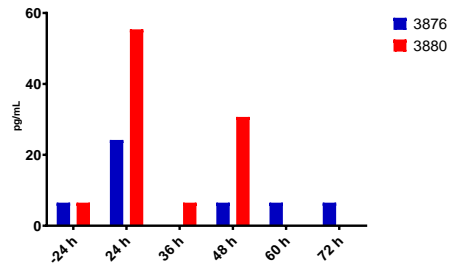

IL-6

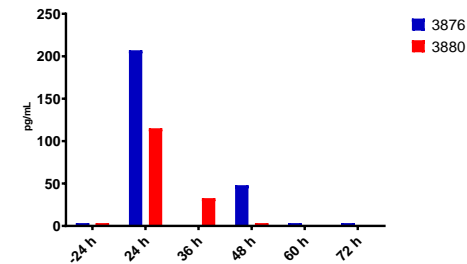

IL-10

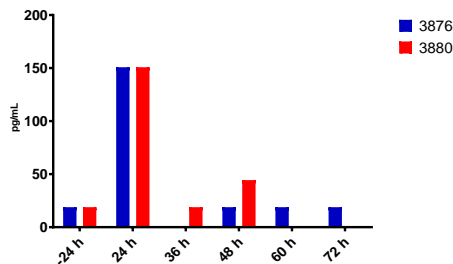

IL-17

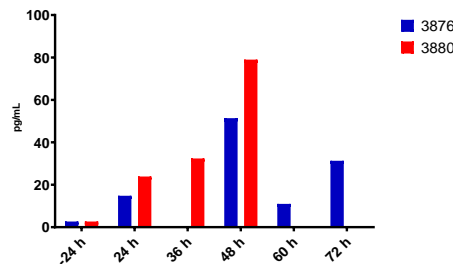IFN- $\gamma$ 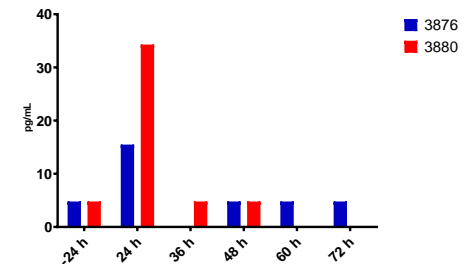

KC

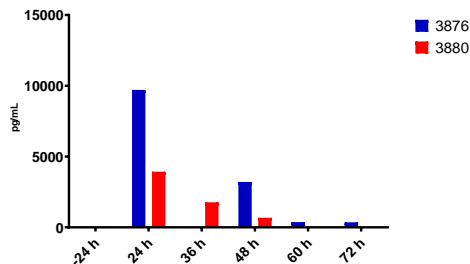

MCP-1

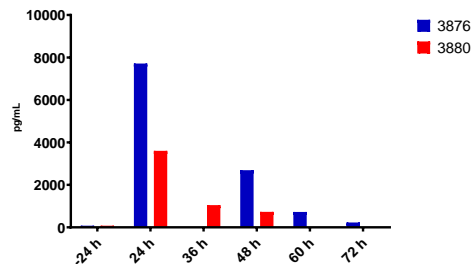TNF- $\alpha$ 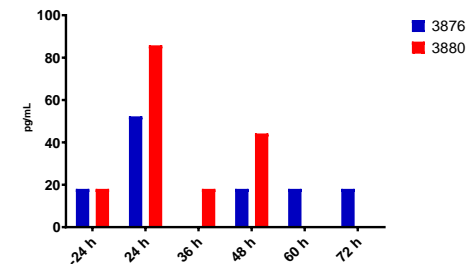

Supplement: Supplementary file 1 [file microorganisms-11-00627-s001.zip › Supplementary-Figure-S6.pdf]
